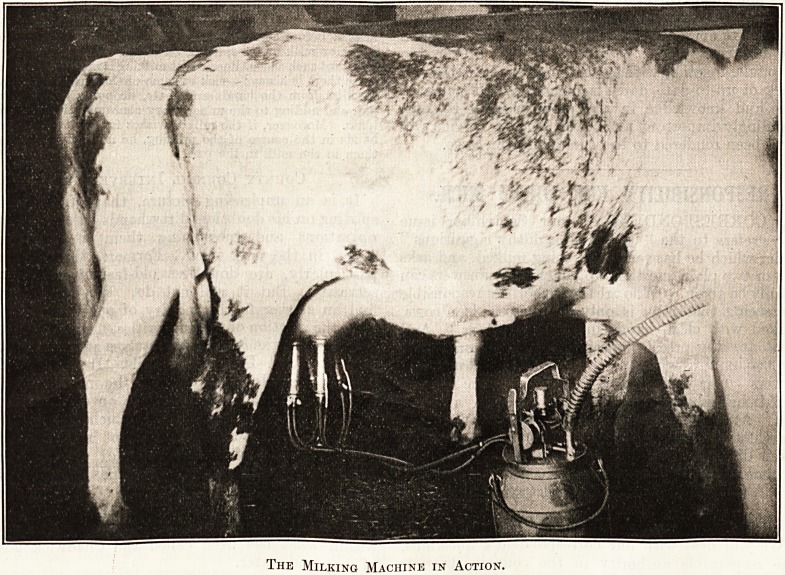# Hospitals and Their Milk Supply

**Published:** 1923-10

**Authors:** 


### HOSPITALS AND THEIR MILK SUPPLY.

### AN IDEAL METHOD.

(By Our Special Correspondent.)

THERE is too much anonymity about the average hospital milk supply. Here and there, no doubt, the wholesaler arranges for the identical churns of milk of a particular farm to proceed direct to a particular hospital. But in the smaller institutions, at any rate, the milk is bulk milk and passes (sometimes too literally) through too many hands before it reaches the patient. The ideal system is for each hospital to know its farm and to be in the position to follow the milk through all its adventures from the cow's udder to the ward milk can. Two hospitals in London are at the present time managing their milk supply on these lines, and the system works so smoothly and satisfactorily that a return to the ordinary method of buying seems, to speak as cautiously as possible, unlikely. The hospitals concerned are neighbours—the Hospital for Sick Children in Great Ormond Street and the National Hospital for the Paralysed and Epileptic in Queen Square. The farm from which the milk is taken is West Hill House, Culworth, Banbury. The cost is the same as if buying were carried out through the usual channels. The extra quality and cleanliness of the milk are a clear gain to the hospitals.

Milk Room at the Hospital for Sick Children.

### A Model Farm.

At West Hill House Mr. A. E. Cooke farms approximately 400 acres. His extensive buildings are a model, especially the part of them devoted to milk production. It is Mr. Cooke's ambition that the whole of the farm, all that is grown on it, all the people who live and work on it, all the beasts and general stock shall be eventually in the service of hospitals and employed producing milk and other produce required daily in these institutions. At present he has 80 cows and heifers and will shortly own 100. They are all young—Mr. Cooke believes in young beasts for milk production and sells his cows always after the birth of the third calf. For in the nature of things young cattle are more healthy and, therefore, likely to yield more and better milk. Of course many of the heifers and cows are not producing, but those "on duty," to use a hospital phrase, are driven to the byres and range themselves obediently with their heads quickly in the feeding trough, replenished from a long passage behind. To see them munch at the mixture of corn, cocoanut cake, etc., one would hardly realise that they had been eating all day; but this constant scientific feeding, whether they are yielding or not, means rich good milk, and increased weight and strength to many a puny hospital patient.

### The Milking Machine.

The necessity of personal cleanliness is impressed upon the men engaged in milking. The cows' udders are first cleansed and then the suckers of the milking machine are applied. The machine in use is the "Vaccar," but most suction machines are the same in principle and a general description may be of service. Suction is developed by a vacuum pump, either single or double acting, worked by any of the usual sources of power on a farm—water, steam, oil, petrol, or electricity. A vacuum of 14 in. to 19 in. is thus obtained, and is transmitted to the byres through pipes running the length of the byre over the head of the cows. Between each pair of cows is either a single or double stall-cock, to which can be attached a flexible tube, the lower end of is connected to the actual milking machine, standing between the cows, which is, of course, movable from stall to stall. The suction actuates a pulsator, which by a piston-valve produces an alternate suction and pause, or rest, thus resembling the intermittent sucking of a calf. This pulsator rests on the top or mouth of the milk-can or receiver, which stands between the pair of cows, and is tightly sealed by the action of the partial vacuum within. The pulsator is usually connected by two rubber tubes—one for the suction, the other for the milk—to a junction or claw, from which lead four or eight short tubes to the teat-cups—that is, an air tube and a milk tube to each teat-cup.

### How It Works.

The cups are made of a suitable metal chosen for ease in cleaning. As a rule they have an indiarubber lining, and when the vacuum acts on the interior of the teat-cup and air is admitted between the rubber lining and outer wall, the rubber contracts, grips the teat, pressing out the milk, which is drawn into the bucket by suction. A little air is also often admitted into the milk tube to aid the flow of the milk. With one or two small technical variations the "Vacca" machine in use at West Hill House answers to this general description, taken from a report made to the Highland and Agricultural Society of Scotland by Mr. William Burkitt, B.Sc., who has made this subject his own. The milk receptacle is sealed until the individual cow is finished with. Its contents are then for convenience poured into a larger vessel, through a strainer. The milk then passes through the cooling apparatus (cooled by well water) direct into the churn, which is hermetically sealed and kept in a cold stone-flagged dairy until removal to the station for despatch to the hospitals. Popular ideas of machine milking are quaint. We have heard of the suction apparatus "drawing blood." To anyone who has seen it, has felt the suction, this is obviously absurd. Cows used to this form of milking will never stand hand milking, and because of this everything must be made in duplicate in case one set of apparatus goes wrong, otherwise the dairy farmer would be in a difficult position.

### Uncontaminated Churns.

A word as to the churns. These are the property of the hospital and have the name of the sender and the receiver upon brass plates on the lids and sides. The air-tight lid cannot be taken off on the journey because the use of special keys is required for the purpose. The churn, with its contents untouched by hand, journeys without fear of contamination to the Marylebone Station, where it is met by a carrier engaged by the hospitals and brought by him into the hospital dairies. Here the churns are opened by the special keys. Mr. Cooke's farm may be inspected by representatives of the hospitals at any time. An examination of the cows by a veterinary

The Milking Machine in Action.

surgeon can always be arranged and his certificate sent for the information of the hospitals.

Periodical Examination of Milk.

At the Hospital for Sick Children the milk intended for the patients is pasteurized. The bottles are washed overnight, and sterilized for 20 minutes before use. When they have been filled with milk by automatic fillers the stoppers and rings are fixed on lightly. The bottles are then put in the sterilizer, the thermometer is placed in position, and the steam is turned on. When the steam registers 176 this temperature is maintained for 15 minutes before the lid of the sterilizer is taken off. The stoppers of the bottles are then fastened down tightly and hot and cold water turned on in a special way for gradual cooling. The bottles filled with milk are next kept in cold water until quite cold, and lastly put into wire baskets and sent to the wards. Some milk is kept unpasteurized for the use of the staff. At the National Hospital for the Paralysed and Epileptic, where there are but few children, no milk is sterilized. The milk in bulk is poured into large porcelain receptacles, specially manufactured in order that every part of them can be kept scrupulously clean. Here it remains in the Milk Room until required for use. But the hospitals are not satisfied by these extensive arrangements. From time to time the milk is independently examined for quality by the respective pharmacists, and a bacteriological examination is made in the laboratory of the Great Ormond Street Institution. By the several processes employed the presence in a harmful form of any organism would without doubt be detected. Further, the cleansing of the churns is an important matter. This must be carried out by the best means available —by high pressure steam for choice—as soon as the churns are empty and before the milk can dry upon the surfaces. If the progressive action of the two hospitals, together with the thorough and enlightened work of up-to-date dairy farmers, leads to increased care and knowledge in the production of an outstandingly important article of food, a service will have been rendered to the community at large.

## Figures and Tables

**Figure f1:**
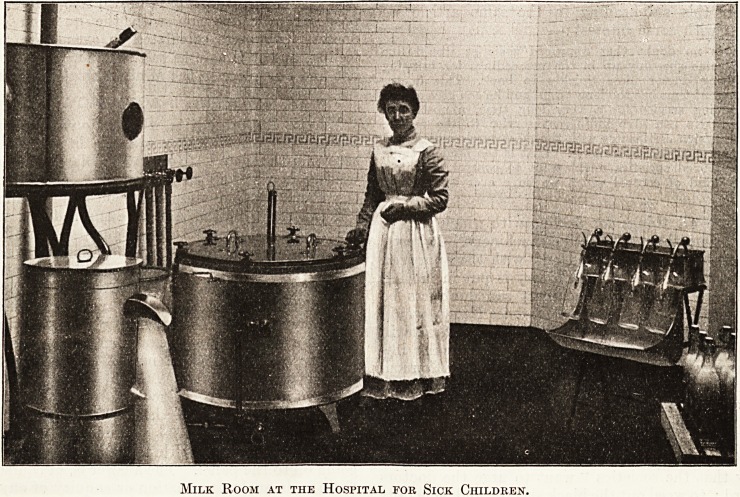


**Figure f2:**